# Recognition of eye diseases based on deep neural networks for transfer learning and improved D-S evidence theory

**DOI:** 10.1186/s12880-023-01176-2

**Published:** 2024-01-18

**Authors:** Fanyu Du, Lishuai Zhao, Hui Luo, Qijia Xing, Jun Wu, Yuanzhong Zhu, Wansong Xu, Wenjing He, Jianfang Wu

**Affiliations:** 1 School of Medical Imaging, North Sichuan Medical College, Nanchong, 637000 China; 2 Faculty of Data Science, City University of Macau, Macau, 999078 China; 3 Guangdong Provincial Key Laboratory of Robotics and Intelligent System, Shenzhen Institutes of Advanced Technology, Chinese Academy of Sciences, Shenzhen, 518000 China; 4 Affiliated Hospital of North Sichuan Medical College, Nanchong, 637000 China; 5 School of Information and Management, Guangxi Medical University, Nanning, 530021 China

**Keywords:** Ocular disease, Computer vision, Deep neural networks, D-S evidence theory, Transfer learning

## Abstract

**Background:**

Human vision has inspired significant advancements in computer vision, yet the human eye is prone to various silent eye diseases. With the advent of deep learning, computer vision for detecting human eye diseases has gained prominence, but most studies have focused only on a limited number of eye diseases.

**Results:**

Our model demonstrated a reduction in inherent bias and enhanced robustness. The fused network achieved an Accuracy of 0.9237, Kappa of 0.878, F1 Score of 0.914 (95% CI [0.875–0.954]), Precision of 0.945 (95% CI [0.928–0.963]), Recall of 0.89 (95% CI [0.821–0.958]), and an AUC value of ROC at 0.987. These metrics are notably higher than those of comparable studies.

**Conclusions:**

Our deep neural network-based model exhibited improvements in eye disease recognition metrics over models from peer research, highlighting its potential application in this field.

**Methods:**

In deep learning-based eye recognition, to improve the learning efficiency of the model, we train and fine-tune the network by transfer learning. In order to eliminate the decision bias of the models and improve the credibility of the decisions, we propose a model decision fusion method based on the D-S theory. However, D-S theory is an incomplete and conflicting theory, we improve and eliminate the existed paradoxes, propose the improved D-S evidence theory(ID-SET), and apply it to the decision fusion of eye disease recognition models.

## Introduction

**Fig. 1:**
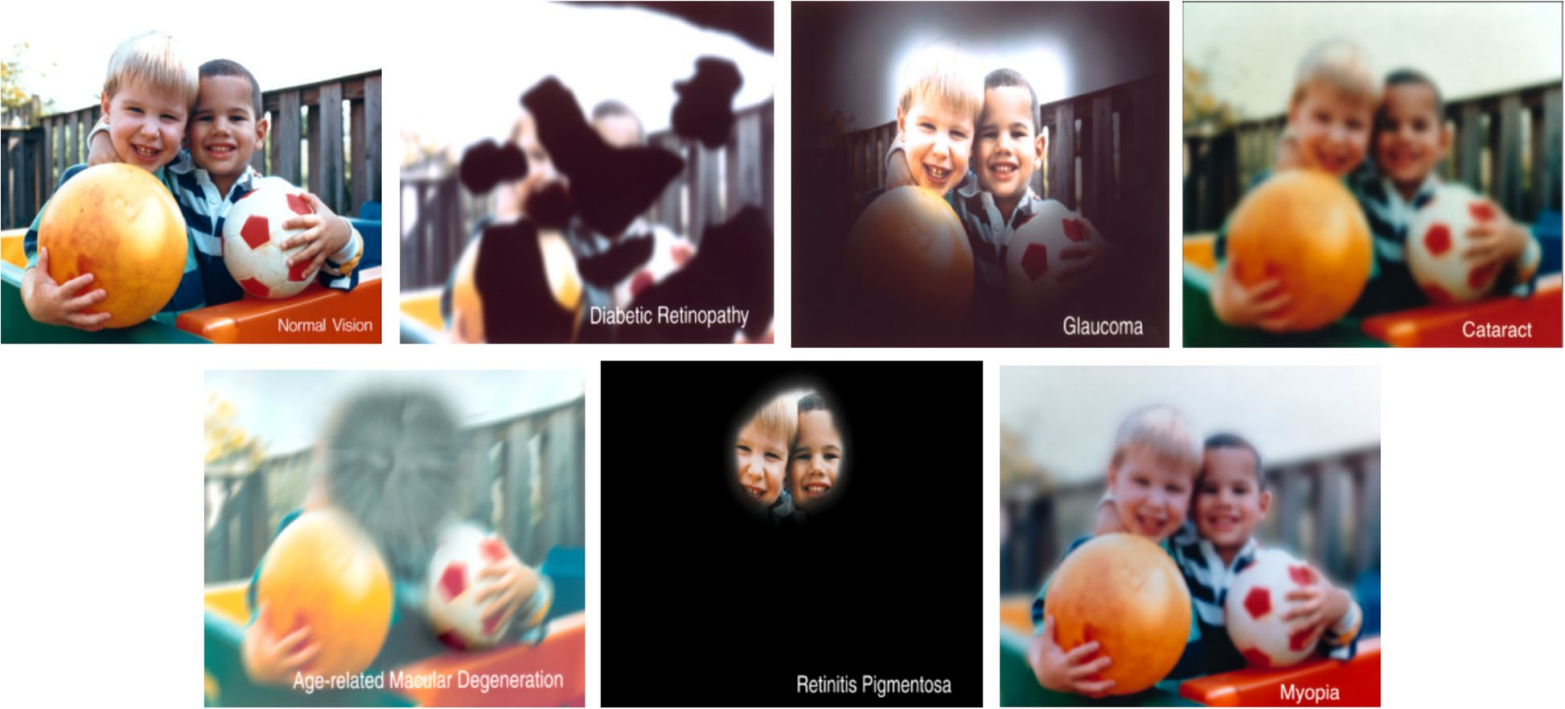
Visual simulation of normal vision alongside common eye diseases. In diabetic retinopathy, black patches obstruct vision. Glaucoma is characterized by a darkening peripheral field while maintaining central vision. In AMD, central vision is impaired, whereas peripheral vision remains intact. Retinitis pigmentosa leads to a complete loss of the visual field around the periphery, with only central vision preserved. Cataracts and myopia both result in blurred vision, but there is a difference between the two. The entire field of view of the cataract is completely blurred, and the field of view of myopia is partially clear [7]. The images are provided by the National Eye Institute (NEI) and are publicly available (https://medialibrary.nei.nih.gov/search?keywords=&f%5B0%5D=category%3A8)

The human eye, the most relied upon of the five senses, processes over 80% of external information through vision. With its unique capabilities, the human visual system excels in classification, detection, and recognition. Recent advancements in computer vision, inspired by biological vision systems, have bridged the gap between biological and computer vision research, particularly through the functional analysis of deep hierarchical structures in primate visual systems [1]. However, individuals may suffer from various eye diseases that impair their vision, and in severe cases, these conditions may even lead to complete vision loss [2], such as glaucoma, often referred to as the thief of human vision. A study reported that by 2013, 64.3 million people aged 40 and 80 had glaucoma, and estimates suggested this figure would rise to 76 million by 2020, and further to 111.8 million by 2040 [3]. Other eye diseases include cataracts, diabetic retinopathy, AMD, myopia, and hypertensive retinopathy. The National Eye Institute conducted simulated experiments to illustrate the vision of individuals with these conditions [4, 5], as depicted [6] in Fig. 1. The World Health Organization emphasizes the early detection of eye diseases as crucial for preventing and treating visual impairment and blindness, affecting 2.2 billion people globally [8, 9]. The human visual system is essential, yet eye diseases often progress unnoticed, and their detection can be complex and time-consuming. With the advancements in computer vision, mirroring human vision, we can apply this technology to detect eye diseases. Prompt detection is vital, and color fundus photographs are preferred in eye disease screening for their effectiveness and affordability [10]. With advances in computer-aided technology, deep neural networks (DNNs) are increasingly utilized in diagnosing eye diseases, exhibiting high accuracy in identifying individual conditions through color fundus photographs, thus serving as valuable tools for medical professionals. Furthermore, it has been demonstrated that existing deep learning models surpass medical personnel in medical image recognition [4, 11, 12].

Deep learning (DL), a subfield of machine learning, is extensively applied in artificial intelligence [13]. Among its most effective techniques is the convolutional neural network (CNN), which excels in automatic feature extraction and learning [14, 15]. CNN employs convolution kernels to analyze images in small perceptual fields, significantly reducing computational demands. Unlike fully connected neural networks, CNNs train only filter weights, which are reused. This efficiency allows for deeper neural networks and more intricate tasks. Perceptual fields enable the inference, perception, and generalization of high-level features like texture, structure, and gradients, leading to enhanced accuracy in image detection, classification, and clinical image classification based on disease conditions [16]. Different eye diseases cause distinct alterations in the retinal nerve fiber layer, making them identifiable through the texture features of retinal fundus images [17], thus rendering CNNs suitable for feature extraction from these images. CNNs create sparse connections through weight sharing and local connectivity, drastically reducing parameter count and harnessing local correlations between adjacent-layer neurons. Modern deep neural networks (DNNs) further deepen CNNs by layering convolution layers, as seen in architectures like VGGNet [18], ResNet [19], and GoogLeNet [20–22]. DNNs have demonstrated significant potential in various applications, notably in image classification and speech recognition [23, 24]. DL demands substantial computing memory and power., necessitating large data sets and graphics processing units (GPUs). While GPUs are generally accessible, acquiring extensive labeled data can be costly, requiring significant financial and material resources. To address these challenges, researchers have adopted “transfer learning” (TL). TL enables the application of previously acquired knowledge to new tasks, substantially reducing training time and lessening the dependency on large data volumes.

For eye disease recognition, Aamir et al. utilized multilevel deep neural networks to classify four states of glaucoma, employing two CNNs: one to distinguish between normal and glaucomatous eyes, and another to categorize glaucoma into advanced, moderate, and early stages [25]. Dinç et al. demonstrated exceptional performance in glaucoma detection using local convolution [26]. The AG-CNN model by Li et al. is currently the most advanced in glaucoma detection and pathologic region localization [27]. Thakoor et al. applied an OCT-based CNN with transfer learning for glaucoma identification [28]. He et al. developed the AUB-Net to recognize eight eye diseases on the ODIR-5 K dataset, uniquely addressing multiple eye diseases concurrently, incorporating left and right eye attention mechanisms, unlike other methods that focus on a single disease [10]. Similarly, Sun et al. introduced AEye Doctor, an automated diagnostic system based on ODIR-5 K, enhancing diagnostic precision with patient interaction and an adjustable saliency heatmap [29], which underscores key areas in retinal images for diagnosis [30]. Zhou et al. implemented an inductive transfer learning approach with a multiscale transfer (MTC) for improved feature extraction, and a domain-specific adversarial adaptation (DSAA) module, balancing disease differentiation and adaptation to target and source data distributions [31].

In our research, we utilize deep neural networks for transfer learning and an enhanced D-S evidence theory to recognize eye diseases. Given that we focused on seven classes of diseases with overlapping characteristics, and considering the escalating complexity in performance enhancement as the number of diseases increases [29], we use ResNet50[18]and ResNet101 [19] as subnetworks for transfer learning. These form classification networks, serving as two basic probability assignment functions *m*_1_, *m*_2_, respectively. Ultimately, we use ID-SET for evidence fusion to obtain the final recognition results. The specific contributions are as detailed follows.

1. We incorporate non-negative monotone softmax functions into D-S evidence theory, resolving the four inherent paradoxes in D-S theory. We introduce an improved D-S evidence theory (ID-SET) and apply it to decision fusion within deep neural networks.
2. To enhance model learning and convergence, we integrate an image enhancement strategy and transfer learning with ResNet models of varying depths. These models are used to identify different eye diseases, applying the improved D-S theory to the decision fusion of the two models.
3. Experimental evaluation demonstrate that our model fusion strategy notably enhances accuracy, thereby validating the effectiveness of our proposed approach.

This paper is organized as follows: Section 1 offers an introductory overview, outlining the research questions and current study status; Section 2 describes our research methodology; Section 3 discusses relevant data; Section 4 details the experiments and result analysis; and Section 5 provides a comprehensive discussion and conclusion.

## Material and methods

### D-S evidence theory

In the context of mathematical and uncertainty theories, D-S evidence theory presents advantages over Bayesian theory due to its ability to handle uncertain and unknown information under less stringent conditions. Compared to traditional probability theory, D-S evidence theory demonstrates superior performance in data fusion-based classification and is extensively applied in domains such as fault diagnosis [32, 33], engineering technology [34], target recognition and tracking [35, 36], and information fusion [37].

The D-S evidence theory framework operates a set Θ = {*A*_1_, *A*_2_, ⋯, *A*_*φ*_}, *A*_*i*_ = (*i* ∈ [1, *φ*], *φ* <  + ∞) denotes a proposition or hypothesis, Θ is called the recognition framework, *A*_1_, *A*_2_, ⋯, *A*_*φ*_ are independent of each other, and the mapping function *m* : 2^Θ^ → [0, 1] is called the basic probability assignment function and satisfies the following equation.

1 $$\left\{\begin{array}{c}m\left(\varphi \right)=0\\ {}m(A)\in \left[0,1\right],\forall A\subset \Theta \\ {}\sum \limits_{A\subset \Theta}m(A)=1\end{array}\right.$$

D-S evidence theory provides a robust method for evidence fusion, integrating evidence from multiple sources. For proposition *A* ⊂ Θ, in the recognition framework Θ, there are a finite number of basic probability assignment functions *m*_1_, *m*_2_, *m*_3_, …*m*_*l*_. The fusion formula is defined as follows:

2 $$\left({m}_1\oplus {m}_2\dots \oplus {m}_l\right)(A)={}^{1}\!\left/ \!{}_{\left(1-k\right)}\right.\times \sum \limits_{A_1\cap {A}_2\dots {A}_{\phi }=A}{m}_1\left({A}_1\right){m}_2\left({A}_2\right)\dots {m}_l\left({A}_{\phi}\right)$$

where:

3 $$k=\sum \limits_{A_1\cap {A}_2\dots {A}_{\phi }=\varphi }{m}_1\left({A}_1\right){m}_2\left({A}_2\right)\dots {m}_l\left({A}_{\phi}\right)=1-\sum \limits_{A_1\cap {A}_2\dots {A}_{\phi}\ne \varphi }{m}_1\left({A}_1\right){m}_2\left({A}_2\right)\dots {m}_l\left({A}_{\phi}\right)$$

*k *represents the conflict factor, which indicates the level to which the evidence contradicts each other, and (1 − *k*) is the normalization coefficient.

The traditional D-S theory is an effective evidence fusion theory, but it will fail under certain circumstances. For example, when the conflict factor *k* → 1, it will fail. There are four typical paradoxes: complete conflict paradox, 0 trust paradox, 1 trust paradox, and high conflict paradox [38]. As shown in Table 1, these four paradoxes are D-S theory failure conditions. In Table 1, *m*_1_, *m*_2_, *m*_3_, *m*_4_, *m*_5_ are the basic probability assignment functions, and the propositions *F*, *G*, *H*, *I*, *J* ⊂ Θ.

**Table 1:** BPA for four typical common paradoxes

Paradoxes	Evidence	Propositions				
		F	G	H	I	J
Complete Conflict paradox	*m*_1_	1	0	0	\	\
	*m*_2_	0	1	0	\	\
	*m*_3_	0.8	0.1	0.1	\	\
	*m*_4_	0.8	0.1	0.1	\	\
0 trust paradox	*m*_1_	0.5	0.2	0.3	\	\
	*m*_2_	0.5	0.2	0.3	\	\
	*m*_3_	0	0.9	0.1	\	\
	*m*_4_	0.5	0.2	0.3	\	\
1 trust paradox	*m*_1_	0.9	0.1	0	\	\
	*m*_2_	0	0.1	0.9	\	\
	*m*_3_	0.1	0.15	0.75	\	\
	*m*_4_	0.1	0.15	0.75	\	\
High conflict paradox	*m*_1_	0.7	0.1	0.1	0	0.1
	*m*_2_	0	0.5	0.2	0.1	0.2
	*m*_3_	0.6	0.1	0.15	0	0.15
	*m*_4_	0.55	0.1	0.1	0.15	0.1
	*m*_5_	0.6	0.1	0.2	0	0.1

In the identified four paradoxes, *k* = 1 is determined in the completely conflict paradox, resulting in a zero denominator. Consequently, the D-S fusion rule ibecomes entirely ineffective. *k* = 0.99 is also determined in the 0 trust paradox, apply (2)(3), and the fusion result is as follows:

4 $${\displaystyle \begin{array}{c}m(F)=0\\ {}m(G)=0.73\\ {}m(H)=0.27\end{array}}$$

Since *m*_3_(*F*) = 0, resulting in *m*(*F*) = 0, no matter the strength of other supporting evidence, the final outcome for the proposition *F* is 0. This shows that the fusion rule has the defect of one-vote veto. *k* = 0.9998 is calculated in the 1 trust paradox, and the fusion result is:

5 $${\displaystyle \begin{array}{c}m(F)=0\\ {}m(G)=1\\ {}m(H)=0\end{array}}$$

Despite all basic probability assignment functions assigning the proposition *G* a small BPA, the final fusion result considers *G* to be a correct proposition. Clearly, this outcome is illogical and impractical for engineering applications. *k* = 0.99986 is calculated in the high conflict paradox, and the fusion result is:

6 $${\displaystyle \begin{array}{c}m(F)=0\\ {}m(G)=0.3571\\ {}m(H)=0.4286\\ {}m(I)=0\\ {}m(J)=0.2143\end{array}}$$

The basic probability assignment functions *m*_1_, *m*_3_, *m*_4_ and *m*_5_ all give proposition *F* a large BPA, the final result inaccurately dismisses the proposition *F* as incorrect. This indicates that highly conflicting evidence can lead to erroneous conclusions.

Due to *k* → 1 and the high conflict among BPAs, D-S theory proves inadequate for evidence fusion. The essential reason is that a certain *BPA* → 0 or the distance between BPAs is too large, and the conflict is high. To address this issue, we improve the D-S theory.

### Improved D-S evidence theory (ID-SET)

Because *BPA* → 0 or the distance between BPAs is too large, the D-S theory becomes ineffective for evidence fusion in the face of high conflict. To address this limitation, various researchers have proposed different fusion rules [39–41], with most methods addressing the issue by modifying the fusion rules.

Our proposed method aims to mitigate the conflict by altering the dimension of *BPAs*. We map *BPAs* to another dimension, effectively reducing the distance between them, ensuring ∀*BPA* > 0 but without altering their comparative magnitudes. For this, we found an exponential function *f*(*x*) = exp(*x*) because it is an increasing function and *f*(*x*) > 0, it meets our requirements, but we know *m*(*A*) ∈ [0, 1], $\sum \limits_{A\subset \Theta}m(A)=1$, exp(*m*(*A*)) ≥ 1, so we have to normalize it as follows.

7 $${m}^{\hbox{'}}\left({A}_{\alpha}\right)={}^{\exp \left(m\left({A}_{\alpha}\right)\right)}\!\left/ \!{}_{\sum \limits_i^{\phi}\exp \left(m\left({A}_i\right)\right)}\right.$$

(7) constitutes the crux of our enhanced algorithm, designed to diminish the distance between *m*(*A*) and make *m*(*A*) ∈ (0, 1) but will not change the size relationship between them, which maintains the validity of (2) because without changing their size relationship, we can still effectively and intuitively select the high probability fusion result when fusing the evidence. Experimental tests reveal that in scenarios where *m*(*A*) = 0, employing (7) successfully resolves the paradox noted in Table 1, as demonstrated in Table 2.

**Table 2:** The ID-SET for BPA

Paradoxes	Evidence	Propositions				
		F	G	H	I	J
Complete Conflict paradox	${m}_1^{\hbox{'}}$	0.576	0.212	0.212	\	\
	${m}_2^{\hbox{'}}$	0.212	0.576	0.212	\	\
	${m}_3^{\hbox{'}}$	0.502	0.249	0.249	\	\
	${m}_4^{\hbox{'}}$	0.502	0.249	0.249	\	\
	*m*^'^	0.748	0.184	0.068	\	\
0 trust paradox	${m}_1^{\hbox{'}}$	0.391	0.289	0.32	\	\
	${m}_2^{\hbox{'}}$	0.391	0.289	0.32	\	\
	${m}_3^{\hbox{'}}$	0.219	0.539	0.242	\	\
	${m}_4^{\hbox{'}}$	0.391	0.289	0.32	\	\
	*m*^'^	0.385	0.382	0.233	\	\
1 trust paradox	${m}_1^{\hbox{'}}$	0.539	0.242	0.219	\	\
	${m}_2^{\hbox{'}}$	0.219	0.242	0.539	\	\
	${m}_3^{\hbox{'}}$	0.252	0.265	0.483	\	\
	${m}_4^{\hbox{'}}$	0.252	0.265	0.483	\	\
	*m*^'^	0.192	0.105	0.703	\	\
High conflict paradox	${m}_1^{\hbox{'}}$	0.3182	0.1746	0.1746	0.1580	0.1746
	${m}_2^{\hbox{'}}$	0.1614	0.2661	0.1971	0.1783	0.1971
	${m}_3^{\hbox{'}}$	0.2915	0.1768	0.1859	0.1599	0.1859
	${m}_4^{\hbox{'}}$	0.279	0.178	0.178	0.187	0.178
	${m}_5^{\hbox{'}}$	0.2914	0.1767	0.1953	0.1599	0.1767
	*m*^'^	0.598	0.127	0.109	0.066	0.099

In summary, the algorithmic framework of our ID-SET is as follows, outlined in Algorithm 1.

**Algorithm 1.**
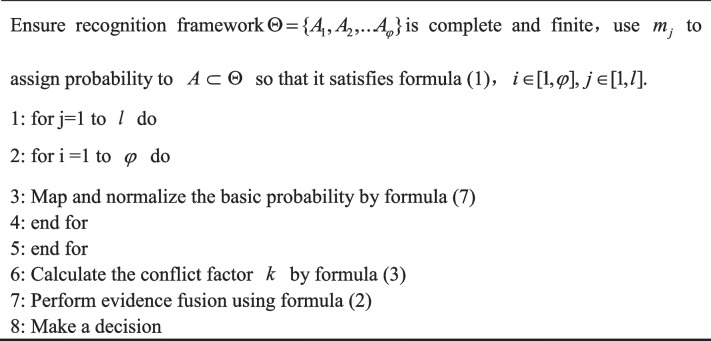
The ID-SET

The values in Table 2 were derived using Algorithm 1 from the data in Table 1. Examination of Table 2 reveals that with the resolution of the complete conflict paradox, *k* = 0.959, the resultant fusion is as follows:

8 $${\displaystyle \begin{array}{c}{m}^{\hbox{'}}(F)=0.748\\ {}{m}^{\hbox{'}}(G)=0.184\\ {}{m}^{\hbox{'}}(H)=0.068\end{array}}$$

Upon fusion, proposition *F* is deemed correct. This outcome aligns with preal-world applications and addresses the issue of the fusion rule becoming invalid when the denominator is zero; following the rectification of the 0 trust paradox, the conflict factor *k* = 0.966, the fusion outcome is:

9 $${\displaystyle \begin{array}{c}{m}^{\hbox{'}}(F)=0.385\\ {}{m}^{\hbox{'}}(G)=0.382\\ {}{m}^{\hbox{'}}(H)=0.233\end{array}}$$

After fusion, the proposition *F* is considered to be the correct proposition, and the result is logical. This method eliminates the defect of one-vote veto. With the resolution of the 1 trust paradox, the conflict factor *k* = 0.961, the fusion result is:

10 $${\displaystyle \begin{array}{c}{m}^{\hbox{'}}(F)=0.192\\ {}{m}^{\hbox{'}}(G)=0.105\\ {}{m}^{\hbox{'}}(H)=0.703\end{array}}$$

The fusion outcome discards the erroneous assertion that proposition *G* is the correct, ultimately determining proposition *H* as the accurate one, which is consistent with practical engineering scenarios; after addressing the high conflict paradox, the conflict factor *k* = 0.998, and the fusion result obtained is:

11 $${\displaystyle \begin{array}{c}{m}^{\hbox{'}}(F)=0.598\\ {}{m}^{\hbox{'}}(G)=0.127\\ {}{m}^{\hbox{'}}(H)=0.109\\ {}{m}^{\hbox{'}}(I)=0.066\\ {}{m}^{\hbox{'}}(J)=0.099\end{array}}$$

The fusion result corrects proposition *F* to be the correct proposition and eliminates the erroneous result caused by the high conflict between the evidence.

Our proposed algorithm’s enhancements effectively eliminate the four prevalent paradoxes in the D-S theory. The improved D-S evidence theory fusion results are logical and in harmony with practical engineering applications, signifying its efficacy as an improvement.

### Overall framework

**Fig. 2:**
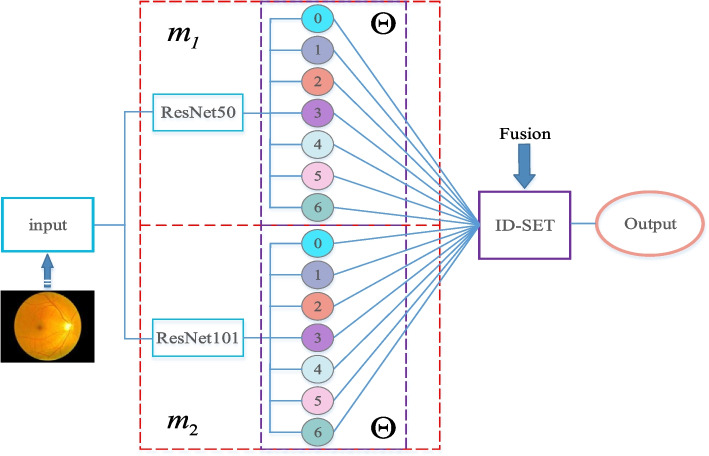
The comprehensive framework for eye disease recognition. Fundus images are used as the common input data. ResNet50 and ResNet101generate BPAs for their respective basic probability assignment functions, which are subsequently fused using our ID-SET to yield the final diagnostic outcomes. The red boxes represent the basic probability assignment function *m*_1_, *m*_2_; The purple boxes are the recognition framework Θ, and the values of its elements correspond to the basic probability assignment BPAs

In this study, we employ DNNs combined with ID-SET to identify 7 classes of fundus images, using ResNet50 [19] as *m*_1_ and ResNet101 [19] as *m*_2_ to generate BPAs. ResNet, recognized as one of the most innovative convolutional neural networks, is selected for its robust fitting capability and ease of implementation. Despite originating from the same architecture, ResNet50 and ResNet101 differ in depth, which translates to varied fitting capabilities and the production of distinct BPAs. While D-S evidence theory is a potent tool for data fusion, its classical D-S evidence theory has the limitation that when a certain *BPA* → 0, it will cause a conflict factor *k* → 1; thus, in this case, traditional D-S evidence theory cannot be applied to evidence fusion. Our work employs the enhanced D-S theory, previously utilized in sensor data fusion in numerous studies [38, 42–44], for the decision fusion of neural network outputs. This decision fusion process is illustrated in Fig. 2.

## Related data

### Introduction of the dataset

**Fig. 3:**
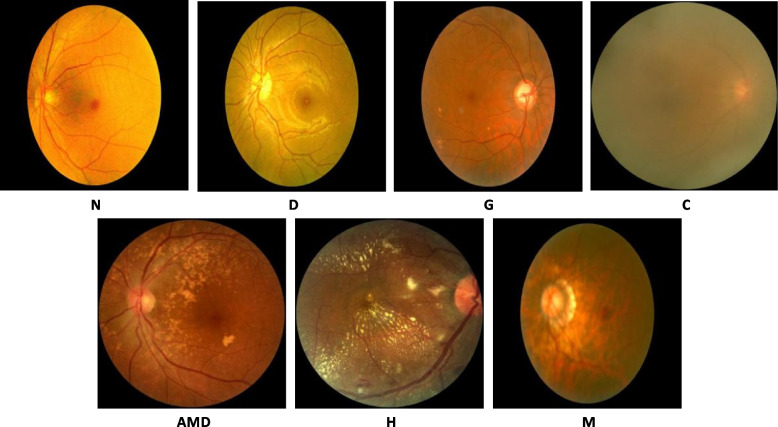
Seven types of fundus images, where N is the normal fundus, D is diabetic retinopathy, G is glaucoma, C is cataract, AMD is age-related macular degeneration, H is hypertension, and M is myopia

The fundus images were sourced from the ODIR-5 K dataset [45], comprising 5000 patients’ details, including color fundus photographs of both eyes and physicians’ diagnostic keywords, collected from various medical institutions in China. This dataset features images captured by different photographic devices, such as Kowa, Zeiss, and Canon. Patient identifiers have been omitted, and descriptions are provided by trained professionals. They categorize eye diseases into eight labels: N, D, G, C, AMD, H, M, and O. Given that ‘O’ is not a specific disease and encompasses multiple conditions [46], we focused on the other seven categories: N, D, G, C, AMD, H, and M. After excluding images of poor quality, those with lens stains, lacking visibility of the optic disc, without fundus photos, with image misalignments, and containing laser spots, a total of 5258 fundus images representing seven types of single eye diseases were selected. The distribution of each type is presented in Table 3, and the characteristics of each type disease category are depicted in Fig. 3.

**Table 3:** The number of fundus images of various types

Eye disease	N	D	G	C	AMD	H	M
number	2818	1385	218	262	237	104	234

### Data augmentation

**Fig. 4:**
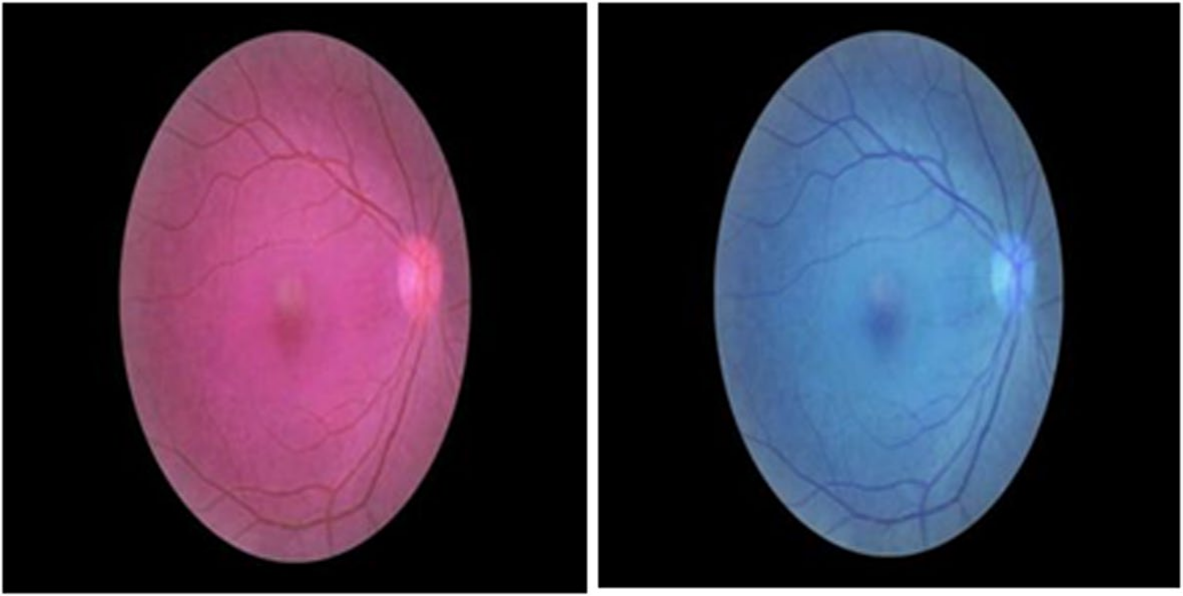
Fundus image after RGB replacement. The left picture is the fundus image of the RBG channel, and the right picture is the fundus image of the BGR channel. The RGB coordinate system is a Cartesian coordinate system. Replacing the order of the channels will not change the structure of the image, but it will change the brightness and contrast of the image, so we use data augmentation

To enhance the dataset’s diversity and minimize the risk of overfitting, we employed data augmentation techniques [16]. Data augmentation helps prevent learning biases caused by the dataset’s limited size and enhances generalization by altering the positions of blood vessel and the optic disc [23, 47]. Moreover, fundus images often contain redundant elements in disease recognition, with pathological areas typically located in or around the optic disc and cup, or adjacent to blood vessels and optic nerves [27, 34]. By resizing images to 512 × 512 × 3 pixels, we removed some redundant content, consequently reducing the computational demands of neural network parameters and shortening processing time. Common data augmentation methods include translation, rotation, cropping, flipping, and label-preserving transformations to increase the number of images [48, 49]. Our approach incorporates random rotation, horizontal and vertical mirroring, and altering the RGB channel sequence to RBG and BGR, effectively expanding the dataset to six times its original size. Post-augmentation, the dataset comprised 31,548 fundus images. Altering the RGB channel order affects the brightness and contrast of the images without changing their structure [50], thus enhancing dataset diversity. We use this method to improve the diversity of the dataset. The fundus image after channel replacement is shown in Fig. 4.

We divided the dataset randomly into a training set and a test set in an 8:2 ratio. The training set includes 25,336 fundus images, and the test set comprises 6212 images. Table 4 displays the classification of the augmented fundus images.

**Table 4:** The number of various fundus images after data augmentation

Eye disease	N	D	G	C	AMD	H	M
Training set	13,588	6665	1046	1260	1138	497	1142
Test set	3320	1645	262	312	284	127	262
Total	16,908	8310	1308	1572	1422	624	1404

## Experiment and results

Our experiment was conducted on a computer equipped with Intel(R) Core(TM) i9-109,200X CPU @ 3.5 GHz, 32G RAM, NVIDIA GeForce RTX 3080 10G GPU. The entire experiment was carried out using Python (version 3.7.9).

**Fig. 5:**
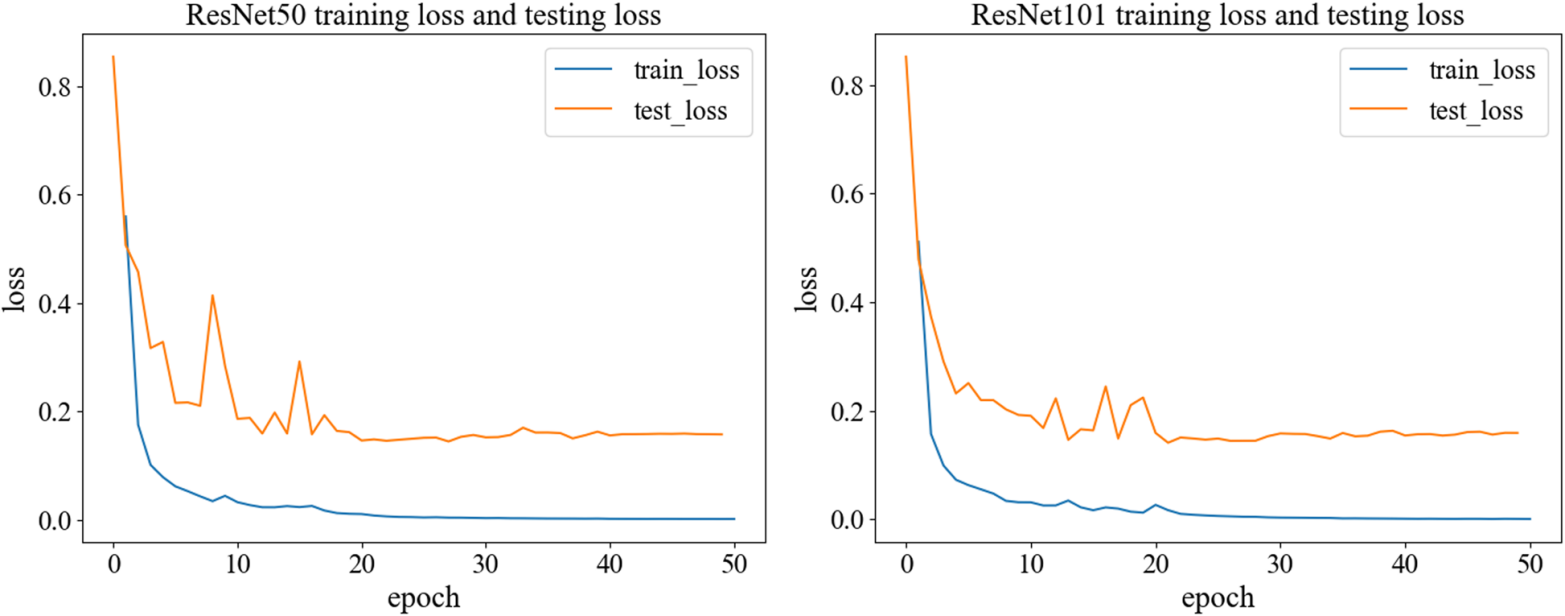
Training loss and test loss. The left panel shows the training loss and test loss for ResNet50. The right panel shows the training loss and test loss for ResNet101

**Fig. 6:**
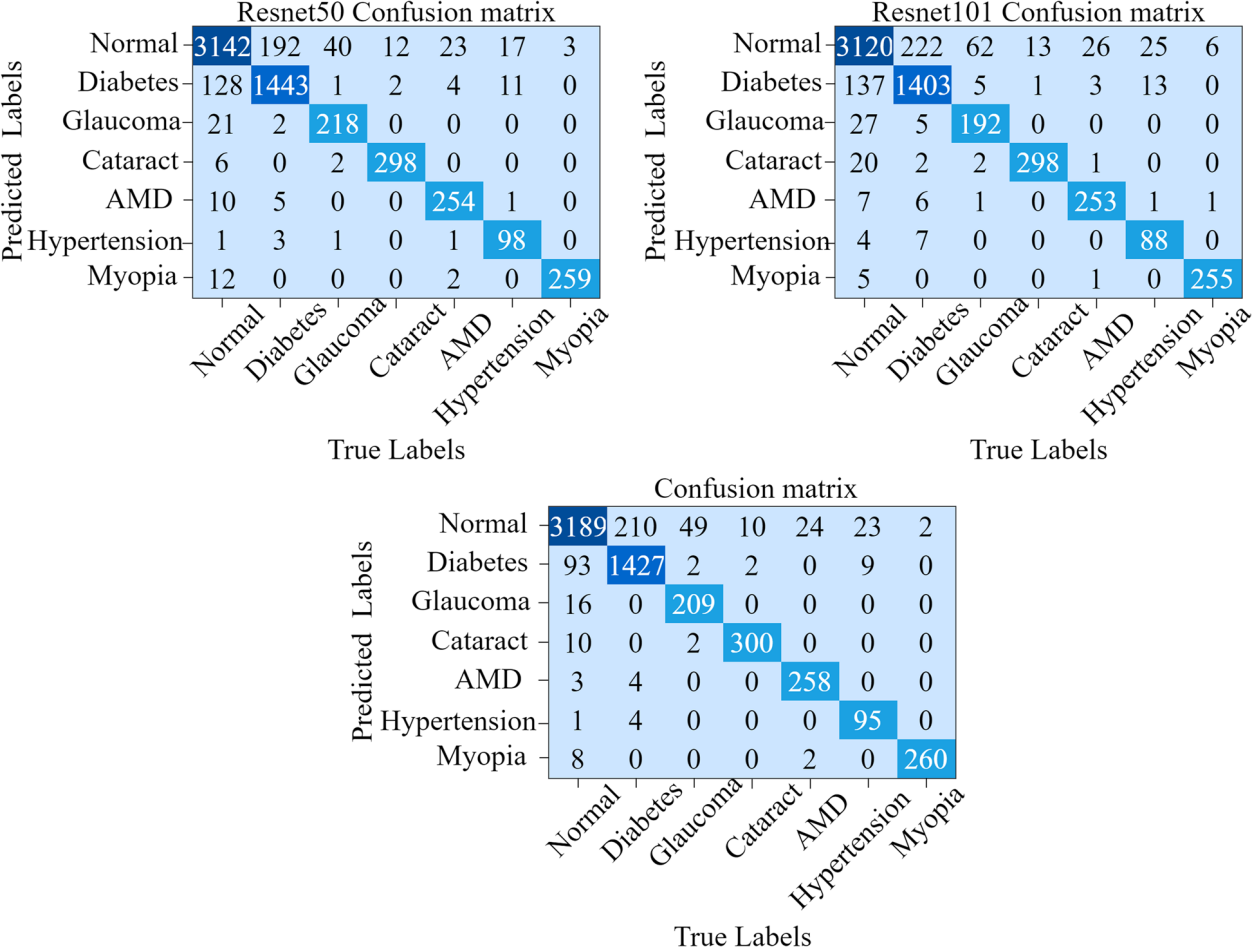
Confusion matrices. The top left, top right and bottom figures are the confusion matrices of ResNet50, ResNet101, and the fusion model, respectively. The test accuracy of the fused model is 92.37%, which is an improvement of 0.46% on ResNet50 and 2.3% on ResNet101

We input the training data into ResNet50 and ResNet101, loaded the pretrained models, and trained them to obtain the basic probability assignment functions *m*_1_, *m*_2_. Both ResNet50 and ResNet101 were trained for 50 epochs, with their corresponding training and testing losses presented in Fig. 5, and the resulting confusion matrices depicted in Fig. 6.

To assess the performance of the proposed model, we evaluated it based on six performance indices: Precision, Recall, Specificity, F1 Score, Kappa coefficient, and the area under the curve (AUC) of the receiver operating characteristic curve (ROC).

12 $${\displaystyle \begin{aligned}Precision&={}^{ TP}\!\left/ \!{}_{\left( TP+ FP\right)}\right.\\ {} Recall&={}^{ TP}\!\left/ \!{}_{\left( TP+ FN\right)}\right.\\ {} Specificity&={}^{ TN}\!\left/ \!{}_{\left( TN+ FP\right)}\right.\\ {}F 1 Score&={}^{ 2\left( Precision\times Recall\right)}\!\left/ \!{}_{\left( Precision+ Recall\right)}\right.\end{aligned}}$$

TP, TN, FP, and FN are the numbers of true-positive samples, true-negative samples, false-positive samples, and false-negative samples, respectively.

**Fig. 7:**
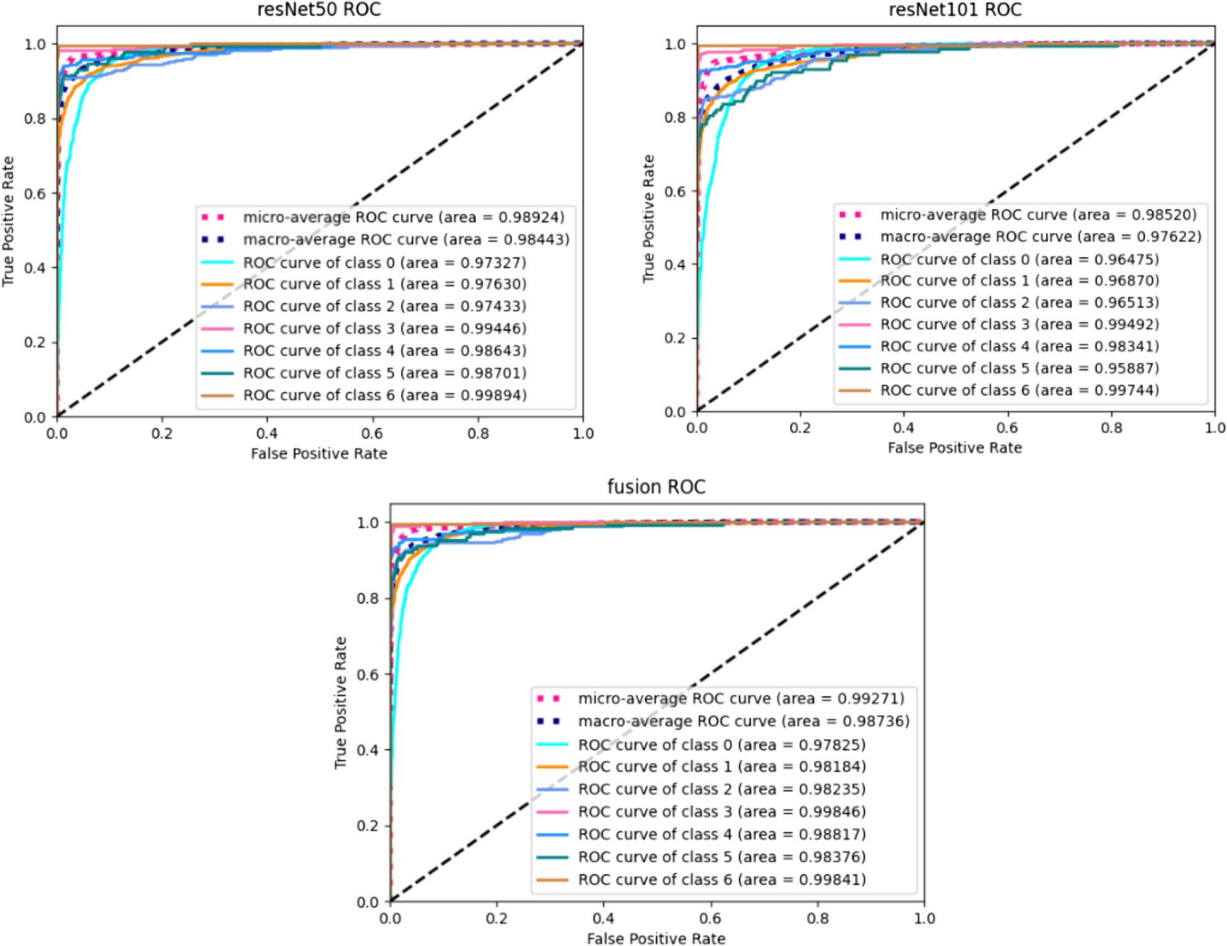
The ROC curves for ResNet50, ResNet101 and the fused model. The two models are fused by D-S theory and the AUC values in the fused model are higher than that of the two independent models

We performed a statistical analysis of each metric at a 95% confidence level. As indicated in Table 5, each metric of the fusion model surpasses the corresponding metric value of the two independent models, demonstrating the efficacy of our proposed method. Furthermore, we plotted the AUC curves for ResNet50, ResNet101, and the fusion model, observing that the AUC area for the fusion model exceeds the respective areas for ResNet50 and ResNet101. These ROC curves are shown in Fig. 7. The ablation analysis in Table 5 and Fig. 7, alongside comparative experiments, confirm that our model fusion approach is effective, with the fused models exhibiting enhanced characterization and decision-making capabilities compared to the individual models.

**Table 5:** Six evaluation metrics for 3 models

Model	%(95% CI)					
	Precision	Recall	Specificity	F1 Score	kappa	Accuracy
ResNet50	93.4(91.5–95.2)	89.5(83.9–95.1)	98(95.3–100)	91.3(87.9–94.7)	0.872	0.915
ResNet101	91.2(88.3–94.1)	86.3(78.1–94.4)	97.5(94.2–100)	88.5(82.9–94)	0.845	0.903
Fusion model	94.5(92.8–96.3)	89(82.1–95.8)	98(95–100)	91.4(87.5–95.4)	0.878	0.9237

Abbreviations: *CI* confidence interval

To further validate our approach, we conducted supplementary experiments on the diabetic retinopathy detection(DRD) dataset [51]. The results, as displayed in Table 6, reveal that the transfer learning-based method surpasses the directly trained method in diabetic retinopathy grade recognition. In this context, ResNet and ViT from [52], which were directly trained, demonstrated lower recognition accuracy compared to our transfer learning-enhanced ResNet. Additionally, both ResNet50 and ResNet101, when based on transfer learning, exhibited lower recognition accuracy than their combined fusion model, further affirming the efficacy of our proposed model.

**Table 6:** Experimental results and comparison on DRD dataset

Paper ID	dataset	Disease labels	Method used	Accuracy
[52]	DRD	No DR, Mild NPDR, Moderate NPDR, Severe NPDR, PDR	ResNet50(Direct training)	0.888
			ResNet101(Direct training)	0.889
			ViT_B_16	0.905
			ViT_L_32	0.914
This paper	DRD	No DR, Mild NPDR, Moderate NPDR, Severe NPDR, PDR	ResNet50(Transfer learning)	0.916
			ResNet101(Transfer learning)	0.922
			ResNet50 + ResNet101 + ID-SET	0.927

In further analysis using the same ODIR-5 K dataset, we compare our work with other researchers’ findings, as illustrated in Table 7. In terms of recognition accuracy and F1 score, our method outperforms most, except for the approach [31]. Across other metrics, our method consistently achieved superior performance. A series of ablation experiments and comparative analyses underscore the effectiveness and potential of our proposed approach, providing valuable insights for multi-model fusion and decision-making processes.

**Table 7:** Comparison of different works under the same dataset

Paper ID	dataset	Disease labels	Method used	Performance
[10]	ODIR-5 K	N,D,G,C,AMD,H,M,O	Attention-based unilateral and bilateral feature weighting and fusion network(AUB-Net)	Kappa: 0.640, F1 Score: 0.913, AUC value: 0.934
[29]	ODIR-5 K	N,D,G,C,AMD,H,M	ResNet	Accuracy: 0.93, Sensitivity: 0.84, Specificity: 0.95, AUC value: 0.90
[46]	ODIR-5 K	N,D,G,C,AMD,H,M,O	Deep CNN	F1 Score: 0.85, Kappa score: 0.31, AUC value: 0.805
[53]	ODIR-5 K	N,C,AMD,M	CNN + 2 Fully Connected Layers	Accuracy: 0.883(95CI (0.812–0.955)) Precision: 0.769(95%CI (0.638–0.901)) Recall: 0.769(95%CI (0.62–0.918)) F1 Score: 0.384(95%CI (0.315–0.454))
			CNN + 5 Fully Connected Layers	Accuracy:0.766 Precision: 0.573(95%CI (0.322–0.825)) Recall: 0.542(95%CI (0.361–0.723)) F1 Score: 0.271(95%CI (0.174–0.368))
[31]	ODIR-5 K	N,D,G,C,AMD,H,M,O	DenseNet+multiscale transfer connection (MTC) + domain-specific adversarial adaptation (DSAA)	Accuracy: 0.945(95%CI (0.904–0.985)) AUC value: 0.938(95%CI (0.928–0.949)) F1 Score: 0.929(95%CI (0.917–0.941)) Kappa: 0.697(95%CI (0.663–0.732))
This paper	ODIR-5 K	N,D,G,C,AMD,H,M	ResNet50 + ResNet101 + ID-SET	Accuracy: 0.9237 Precision: 0.945(95% CI (0.92.8–0.963)) Recall: 0.89(95%CI (0.821–0.958)) Specificity: 0.98(95%CI (0.95–1)) AUC value:0.987 F1 Score: 0.914(95%CI (0.875–0.954)) Kappa: 0.878

## Conclusion

Computer vision is advancing rapidly, yet eye diseases often progress unnoticed. Early detection and treatment are critical for managing these conditions. Recently, DL has emerged as a valuable tool for medical professionals, particularly in fundus image recognition. We proposed a method for recognizing eye diseases using DNNs for transfer learning and ID-SET, focusing on seven types of fundus images within the ODIR-5 K dataset for training and testing. To mitigate the risk of overfitting, we employed data augmentation technology, notably using RGB channel replacement to alter the brightness and contrast of fundus images, effectively increasing the dataset size sixfold. Additionally, we implemented *l*_2_ regularization. The hyperparameter values *λ* for the ResNet50 and ResNet101 models were set at 3e-5, with a learning rate of 5e-4. After loading pretrained models on ResNet50 and ResNest101, we used the two models as *m*_1_ and *m*_2_ to generate their own BPAs, and output the final recognition results after ID-SET fusion. The final results demonstrated an Accuracy of 92.37%, an AUC value of 0.987, an F1 Score of 0.914 (95% CI [0.875–0.954]), and a Kappa coefficient of 0.878, outperforming related work on the same dataset. For future studies on eye diseases, we aim to explore multimodal feature extraction and fusion utilizing D-S theory.

## Data Availability

The ODIR-5 K data that support the findings of this study are openly available at https://www.kaggle.com/datasets/andrewmvd/ocular-disease-recognition-odir5k. The DRD data that support the findings of this study are openly available at https://www.kaggle.com/c/diabetic-retinopathy-detection/data.
